# Cardiopulmonary resuscitation quality and beyond: the need to improve real-time feedback and physiologic monitoring

**DOI:** 10.1186/s13054-016-1371-9

**Published:** 2016-06-28

**Authors:** Steve Lin, Damon C. Scales

**Affiliations:** Rescu, Li Ka Shing Knowledge Institute of St. Michael’s Hospital, Toronto, Ontario Canada; Division of Emergency Medicine, Department of Medicine, University of Toronto, Toronto, Ontario Canada; Institute of Health Policy, Management and Evaluation, University of Toronto, Toronto, Ontario Canada; Department of Critical Care Medicine, Sunnybrook Health Sciences Centre, Toronto, Ontario Canada; Interdepartmental Division of Critical Care Medicine, University of Toronto, Toronto, Ontario Canada

**Keywords:** Randomized controlled trial, Cardiopulmonary resuscitation, Feedback, End-tidal carbon dioxide, Near-infrared spectroscopy

## Abstract

High-quality cardiopulmonary resuscitation (CPR) has been shown to improve survival outcomes after cardiac arrest. The current standard in studies evaluating CPR quality is to measure CPR process measures—for example, chest compression rate, depth, and fraction. Published studies evaluating CPR feedback devices have yielded mixed results. Newer approaches that seek to optimize CPR by measuring physiological endpoints during the resuscitation may lead to individualized patient care and improved patient outcomes.

## Background

Vahedian-Azimi et al. [1] conducted the first in-hospital randomized controlled trial (RCT) evaluating a new cardiopulmonary resuscitation (CPR) feedback device—the Cardio First Angel—in cardiac arrest patients. High-quality CPR has been shown to improve survival outcomes after cardiac arrest [2] and continues to be a focus of quality assurance and improvement programs. There are multiple feedback devices that are commercially available to improve the quality of delivered CPR, but evaluations have yielded mixed results in published RCTs and observational studies [3–6]. Appropriately, international resuscitation guidelines do not currently recommend implementing CPR feedback devices for clinical practice in isolation but rather as part of a comprehensive system of care for cardiac arrest [5, 6].

## Main text

There are several important CPR process measures that currently define high-quality CPR: compression rate (100–120/minute), compression depth (5–6 cm in adults), allowing for complete chest recoil after each compression, and minimizing interruptions in compressions measured by chest compression fraction [5, 6]. These quantitative CPR process measures have been the most common targets of prior evaluations of CPR [3, 4]. Vahedian-Azimi et al. [1] instead developed two checklists to evaluate CPR: an effectiveness score (ranging from 0 to 10) and a guideline adherence score (ranging from 0 to 10). These scores assessed several aspects of CPR quality but a limitation is that assessors were unblinded to treatment allocation, increasing the potential for measurement bias. Overall, the new device improved both measures of CPR quality. The generalizability of these findings is also uncertain, especially considering that rates of cardiac arrest were markedly higher (approximately 35 %) than is typically expected among patients admitted to ICUs in other regions [7].

Chest compressions guided by CPR feedback devices to ensure adequate rate and depth are important [8]; however, their effect on patient physiology remains unclear. Indeed, current resuscitation guidelines use a “one size fits all” strategy, recommending the same compression rate and depth for all patients. Newer approaches to measuring physiology in real time during CPR may improve our ability to evaluate the quality of resuscitation in the future. Physiologic monitoring, specifically cardiac output and coronary and cerebral perfusion during resuscitation, are likely to be more sensitive to small changes that may guide resuscitative efforts, and at least in some studies have been correlated with improved clinical outcomes [9, 10]. However, these measurements are difficult to obtain during real-life cardiac arrest resuscitation, particularly in the out-of-hospital setting. Another area of active investigation is the use of end-tidal carbon dioxide (ETCO_2_) measurements, which are an indirect correlate of cardiac output and are readily available to rescuers performing CPR in cardiac arrest patients; however, as yet there are no recommended ETCO_2_ target values for CPR, and their relationship with survival remains unclear [11]. There is even less evidence for other physiologic measurements; for example, near-infrared spectroscopy to measure cerebral oximetry [12–15].

## Conclusion

As the ability to monitor physiology during cardiac arrest resuscitation improves, we should be able to individualize patient care by performing goal-directed CPR with the ultimate goal of improving patient survival and functional outcomes. This will require focused research into the effectiveness of goal-directed CPR using physiologic monitoring compared to current CPR standards.

## Abbreviations

CPR, cardiopulmonary resuscitation; ETCO_2_, end-tidal carbon dioxide; RCT, randomized controlled trial.
